# SnoRNA U50 Levels Are Regulated by Cell Proliferation and rRNA Transcription

**DOI:** 10.3390/ijms140714923

**Published:** 2013-07-17

**Authors:** Annalisa Pacilli, Claudio Ceccarelli, Davide Treré, Lorenzo Montanaro

**Affiliations:** Department of Experimental, Diagnostic and Specialty Medicine, University of Bologna, Sant’Orsola-Malpighi University Hospital, via Massarenti, 9, Bologna 40138, Italy; E-Mails: annalisa.pacilli@gmail.com (A.P.); claudio.ceccarelli@unibo.it (C.C.); davide.trere@unibo.it (D.T.)

**Keywords:** snoRNA U50, ribosome methylation, IRES-mediated translation, colon cancer

## Abstract

rRNA post transcriptional modifications play a role in cancer development by affecting ribosomal function. In particular, the snoRNA U50, mediating the methylation of C2848 in 28S rRNA, has been suggested as a potential tumor suppressor-like gene playing a role in breast and prostate cancers and B-cell lymphoma. Indeed, we observed the downregulation of U50 in colon cancer cell lines as well as tumors. We then investigated the relationship between U50 and proliferation in lymphocytes stimulated by phytohemagglutinin (PHA) and observed a strong decrease in U50 levels associated with a reduced C2848 methylation. This reduction was due to an alteration of U50 stability and to an increase of its consumption. Indeed, the blockade of ribosome biogenesis induced only an early decrease in U50 followed by a stabilization of U50 levels when ribosome biogenesis was almost completely blocked. Similar results were found with other snoRNAs. Lastly, we observed that U50 modulation affects ribosome efficiency in IRES-mediated translation, demonstrating that changes in the methylation levels of a single specific site on 28S rRNA may alter ribosome function. In conclusion, our results link U50 to the cellular proliferation rate and ribosome biogenesis and these findings may explain why its levels are often greatly reduced in cancers.

## 1. Introduction

Ribosome biogenesis is a highly coordinated process occurring in the nucleolus, where a polycistronic pre-ribosomal RNA (pre-rRNA) transcript is processed to generate the mature 18S, 5.8S, and 28S rRNA. During this processing, the rRNA sequences undergo extensive covalent nucleotide modification, largely directed by small nucleolar RNA (snoRNA)-protein complexes (snoRNP) [1,2]. SnoRNA may be divided into two classes: The H/ACA and the C/D box, mediating pseudouridylation and 2′*O*-methylation of specific sites, respectively [3,4]. In particular, the methylation reaction is guided by an extensive region (10–21 nt) of complementarity between the C/D box snoRNA and rRNA sequences flanking the modification site [5–8]. In mammals, snoRNAs are transcribed by the RNA Polymerase II being localized within the introns of snoRNA host genes. These host genes are also transcribed for either protein coding or noncoding mRNAs [9] which often contain a 5′ terminal oligopyrimidine (5′ TOP) sequence responsible for their translational upregulation in response to growth factors or other conditions requiring increased protein synthesis (reviewed by Meyuhas *et al.* [10]); however, the precise function of 5′ TOP motif with respect to snoRNA synthesis is unknown [11]. Furthermore, the development of three-dimensional maps of the modified nucleotides in the ribosomes of Escherichia coli and yeast has revealed that rRNA modifications occur in conserved and functionally important regions for subunit–subunit and nascent protein interactions, for tRNA and mRNA binding, but not in those interacting with proteins (see [12,13]). This correlation indicates that modifications influence both the structure and the function of the ribosome [14]. Indeed, there is evidence that post-transcriptional rRNA modifications, including pseudouridylation and methylation, affect ribosomal function [15–17] and that alterations in this modification pattern might be involved in human diseases, such as ribosomopathies and tumorigenesis [18,19]. Recent reports have demonstrated that somatic rearrangements, mutations, or the reduction in the expression of the C/D snoRNA U50 have been found in breast carcinomas, prostate cancer, and B-cell lymphomas [20–23]. snoRNA U50 is known to mediate the methylation of ribose residues corresponding to the cytosines in positions 2848 and 2863 in 28S ribosomal RNA [5,23]. In breast cancer cell lines, the reintroduction of U50 is able to induce cell death, suggesting a tumor-suppressor-like behavior for this snoRNA [23]. The human snoRNA U50 sequence is localized in the 5th intron of the non-coding host gene named small nucleolar RNA host gene 5 (SNHG5) [20,24], which is a member of the 5′ TOP gene family.

In this paper we investigated the relationship between snoU50 and cancer in colon cancer cell lines and tumors with particular regard to proliferation.

## 2. Results and Discussion

### 2.1. Evaluation of U50 Levels in Colorectal Cancer Tissues and Cell Lines

**Figure 1:**
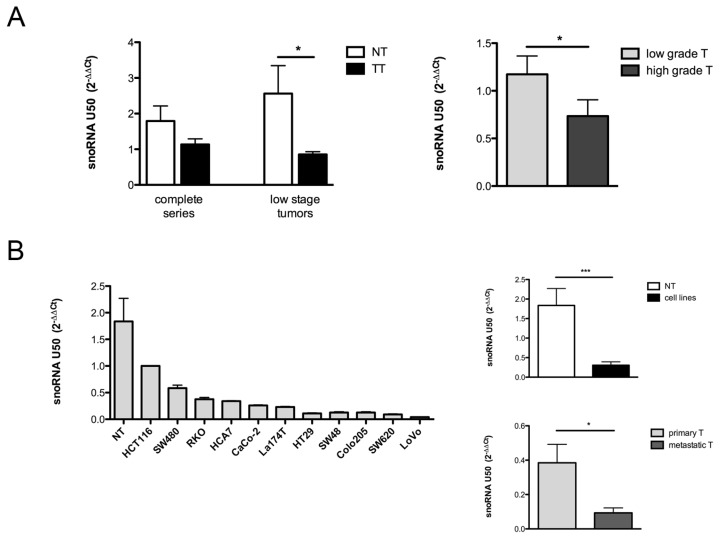
Downregulation of U50 levels in colon cancer tumors and cell lines. (**A**) Evaluation of U50 levels in both tumoral and adjacent untransformed colon tissues on all samples (*n* = 34) and in a subgroup of low-stage tumors (*n* = 20) (left); comparison of U50 levels between low- and high-grade tumors (*n* = 27 and *n* = 7, respectively); (**B**) Evaluation of U50 levels in a cohort of colon cancer cell lines (left); comparison between U50 levels in normal colon tissues and cell lines (right-top) and between cell lines derived from primary or metastatic tumors (right-bottom). The results correspond to means ± S.E.M. of three different experiments. *p ≤ 0.05; ***p ≤ 0.001; NT, normal tissues; TT, tumoral tissues; T, tumors.

The evaluation of snoRNA U50 levels on colon cancer tissues was performed on both tumor and normal tissues in a series of 34 patients. We found that U50 was downregulated in tumor tissues if compared to the normal counterpart and this reduction was statistically significant in a subgroup of low-stage tumors (*p* = 0.047) (Figure 1A, left). The decrease in U50 levels in tumors was in line with previous reports, demonstrating the same behavior in prostate and breast cancers [21,23]. In order to find a key element linking U50 and tumorigenesis, we performed a correlation analysis with the available clinical and bio-pathological features of tumors (see Table S1) and we found a significant association with the tumor grade. Indeed, high-grade tumors displayed lower U50 levels in comparison to those observed in low-grade tumors (*p* = 0.049) (Figure 1A, right). We then evaluated U50 expression in a panel of eleven colon cancer cell lines. We found that U50 expression is highly variable between lines, but always lower than normal colon tissues (NT) (Figure 1B, left), while the overall comparison between NT and colon cancer cell lines showed a statistically significant difference for U50 expression (*p* = 0.0004) (Figure 1B, right-top). Furthermore, we found that the U50 expression in lines derived from primary tumors (HCT, SW480, RKO, HCA7, CaCo-2, La174T, HT29, SW48) was significantly different from that in those derived from metastatic tumors (Colo205, SW620, LoVo-*p* = 0.0121) (Figure 1B, right-bottom).

### 2.2. Relationship between U50 and Proliferation

Compared to untransformed tissues, U50 was downregulated in tumors. Being that one of the major distinguishing characteristics between these tissues is their proliferation activity, we investigated whether there is a relationship between U50 and proliferation.

**Figure 2:**
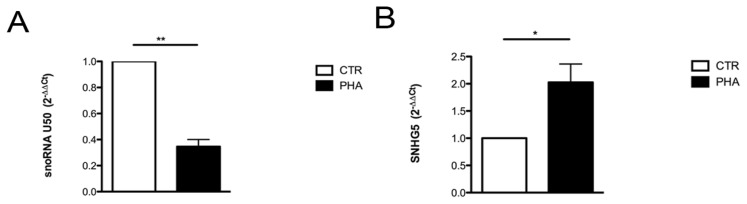
Regulation of U50 levels in response to cellular proliferation. Evaluation of U50 (**A**); and its host gene SNHG5 (**B**) levels in human lymphocytes stimulated or not to proliferate with PHA for 72 h. The results correspond to means ± S.E.M. of four different experiments. *p ≤ 0.05; **p ≤ 0.01. CTR, controls; PHA, phytohemoagglutinin.

**Figure 3:**
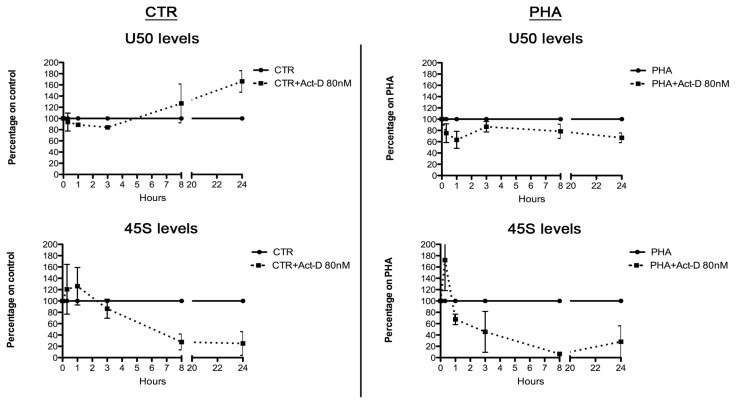
Relationship between U50 and rRNA transcription. Evaluation of U50 and 45S levels in human lymphocytes controls or PHA-stimulated in the presence or absence of high doses of the transcriptional inhibitor Act-D (80 nM) in order to block both polymerases I and II. The analyses were performed 20 min, 1 h, 3 h, 8 h, and 24 h after Act-D treatment. The results correspond to means ± S.E.M. of three different experiments. CTR, controls; PHA, phytohemoagglutinin; Act-D, actinomycin-D.

In order to study changes in U50 synthesis depending on cellular proliferation, we used a well-established model of investigation, *i.e*., the comparison of resting and stimulated primary human lymphocytes. Thus we isolated lymphocytes from healthy donors and performed lymphocyte proliferation assays by using phytohemoagglutinin (PHA). After PHA stimulation, we evaluated the expressions of both U50 and its host gene SNHG5 and observed a strong reduction of U50 levels in PHA stimulated cells, compared to controls (Figure 2A). Conversely, the SNHG5 host gene was upregulated (Figure 2B). Since snoU50 and SNHG5 are simultaneously transcribed, this discrepancy might be explained by a different stability of the two RNAs. The massive decrease in U50 led us to hypothesize that during cellular proliferation U50 stability might be extremely reduced. To prove this hypothesis, we performed mRNA stability assay on lymphocytes—Both stimulated and not stimulated by PHA—By treating cells with the transcriptional inhibitor Actinomycin D (Act-D) at an 80 nM concentration. At this concentration, Act-D is known to abolish the activity of RNA polymerases I and II. We observed that the Act-D treatment did not reduce U50 stability in non-proliferative control cells, but rather induced a progressive accumulation of this snoRNA depending on the length of Act-D treatment (Figure 3). On the contrary, in PHA stimulated cells, we found an early decrease in U50 levels up to 3 h of treatment, followed by a general stabilization of U50 levels (Figure 3). The fluctuation of U50 levels observed at 3 h may be the result of the combined effect of time needed to obtain the full inhibition of ribosome processing by Act-D treatment and the regulation of SNHG5 transcription as documented in Figure S1. Furthermore, PHA treatment induced a notable decrease in U50 levels but an increase in SNHG5 levels. The different levels of our transcripts in the two conditions before Act-D treatment and the behavior of mature SNHG5 mRNA levels observed after 80 nM Act-D treatment excludes the possibility that the increase of U50 levels after treatment was due to an increase of its transcription.

We hypothesized that the observed stabilization of U50 was a consequence of the concomitant transcriptional inhibition of rRNA genes, which implied a reduced post-transcriptional modification of rRNA, and thus a lower U50 consumption. This would be in line with the variation of 45S rRNA levels found in both control and PHA-treated lymphocytes (Figure 3). The different regulation of 45S levels over time observed in control and PHA treated cells can be explained by the different activity of rRNA processing in the two conditions. Taken together, these results showed that U50 and 45S had opposite behaviors. This is particularly clear in control cells, since the ribosome biogenesis is not as intensive and U50 did not have to be massively consumed.

### 2.3. Relationship between U50 and rRNA Transcription

The accumulation of U50 induced by the block of total transcription and the inverse relationship between U50, proliferation, and 45S levels led us to suppose that U50 synthesis could be regulated by the ribosome biogenesis rate, and so we selectively reduced rRNA transcription in SW620 colon cancer cells. We chose this line since the levels of U50 are quite low in presence of a very active ribosome biogenesis activity.

**Figure 4:**
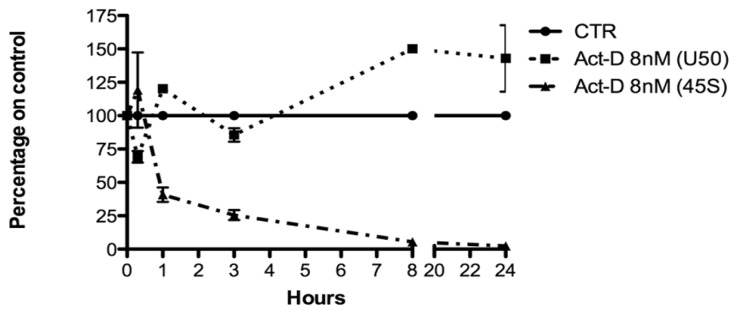
Relationship between U50 and *de novo* rRNA transcription in SW620 cell line. Evaluation of U50 stability performed by measuring U50 and 45S levels in SW620 cells after blockade of Polymerase I with low doses of Act-D. The analyses were performed 20 min, 1 h, 3 h, 8 h, and 24 h after Act-D treatment. The results correspond to means ± S.E.M. of three different experiments. CTR, controls; Act-D, actinomycin-D.

A tenfold lower Act-D concentration was used to selectively inhibit polymerase I only. This selectivity was proved by measuring the transcription levels of a housekeeping mRNA (β-glucuronidase-GUS) and SNHG5, whose genes were both transcribed by polymerase II. Results demonstrated that none were affected by Act-D 8nM treatment (Figure S2). The SNHG5 transcript was also measured to exclude the possibility that U50 levels could be regulated by its transcription. After 45S synthesis inhibition, U50 levels increased (Figure 4). This result obtained after the blockade of rRNA transcription confirmed our hypothesis that U50 and cellular proliferation are inversely associated because of its massive consumption and not because of its transcription. Indeed, U50 is implicated in post-transcriptional methylation of 28S rRNA, and it is well known that during proliferation there is an increase in ribosome biogenesis requiring modification of rRNA molecules. The levels of U50 and 45S transcript displayed opposite behaviors, thus confirming that the strict dependence of U50 on the rRNA transcription rate might be due to its biological function. Taken together, these data indicated a putative role of U50 and snoRNAs in growth, tumorigenicity, and metastasis. We then investigated whether the relationship between ribosome biogenesis and U50 is shared by other snoRNAs and evaluated the cellular levels of 3 additional C/D box snoRNAs such as U33, U34, and U56 housed in genes coding for the ribosomal protein L13A (U33 and U34) and for the nucleolar protein 5A, which is a component of snoRNPs complex (Nop56). We found that these selected snoRNAs displayed the same regulation and that, in all cases, the accumulation started after 3 h and progressively increased with the length of treatment (Figure S3).

### 2.4. Effect of U50 Regulation on rRNA Site-Specific Methylation and Ribosome Activity

**Figure 5:**
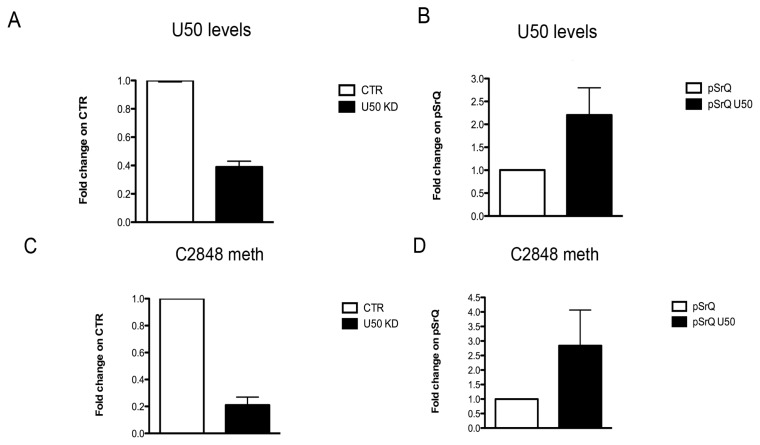

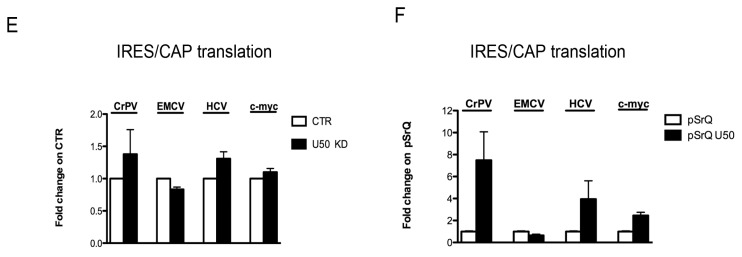
Effect of U50 modulation on C2848 methylation and IRES-mediated translation. Evaluation of U50 levels (**A** and **B**); C2848 methylation status (**C** and **D**); and IRES-mediated translation (**E** and **F**) in HCT116 cells after 72 h of U50 knockdown (left panel) and U50 overexpression (right panel). The results correspond to means ± S.E.M. of three different experiments. CTR, controls; KD, knockdown; pSrQ, pSIREN-RetroQ vector (empty vector); pSrQ U50, pSIREN-RetroQ-U50 vector; CrPV, CRicket Paralysis Virus; EMCV, EncephaloMyoCarditis Virus; HCV, Hepatitis C Virus.

Prompted by these observations, we investigated the possible effect of U50 modulation on mRNA translation. Indeed, U50 is responsible for the site-specific C2848 methylation on 28s rRNA, and It has recently been demonstrated that incorrect methylation of rRNA is associated with an impaired capacity to initiate translation through Internal Ribosome Entry Site (IRES) [19,25] by influencing the mechanism of 80S complex formation on IRES elements [25]. Actually there are studies reporting that changes in snoRNAs are crucial for ribosome biogenesis or function [26,27], and showing an increase in non-coding host genes either after inhibition of translation and elongation [11,28] or in growth arrest conditions [29]. However, it is not clear whether the modulation of a single snoRNA might also contribute to altering ribosome efficiency. In this paper, we focused both on the role of U50 modulation in C2848 site-specific methylation, and on IRES-mediated translation. Therefore, we semi-quantitatively evaluated the changes in C2848 methylation in PHA stimulated cells and observed a decrease in site-specific C2848 methylation in response to proliferation (Figure S4). To better understand the role of U50 and its influence on methylation and IRES-mediated translation, we regulated U50 levels by specific knockdown (KD) and overexpression. We then evaluated the effect on C2848 of 28S methylation, on the translation of known viral IRESes types (Cricket Paralysis Virus-CrPV, Encephalomyocarditis virus-EMCV and Hepatitis C Virus-HCV), and on a cellular IRES (c-Myc) known to be affected by rRNA methylation [25]. We performed U50 KD on HCT116 cell lines and observed that, 72 h after LNA transfection, we obtained a rather strong U50 KD efficiency associated with a decrease in C2848 methylation (Figure 5A,C). Thus we decided to evaluate IRES-mediated translation after 72 h of U50 KD. We found that U50 KD did not significantly alter ribosome activity (Figure S3, top), although we observed a slight increase in CrPV- and HCV IRESes-mediated translation (Figure 5E). It is likely that we could not appreciate notable differences in ribosome translation activity in the U50 KD model because the experiments were performed in a tumor cell line with already low U50 levels in comparision to untransformed cells.

Furthermore, in order to investigate the effect of U50 overexpression, we used the pSrQ-U50 vector to stably transfect HCT116, SW620, and LoVo cell lines. In addition to HCT116, we chose SW620 and LoVo cells since they displayed the lowest U50 basal level in our cohort of colon cancer lines, as previously reported. We successfully obtained all U50 overexpressing lines, but an increase in C2848 methylation was achieved for the HCT116 line only (Figures 5B,D and S6). In contrast with results after U50 KD, in the presence of higher C2848 methylation levels, we found a general decrease in CAP-mediated translation and an increase in IRES-mediated translation (Figure S5, bottom). These observations were consistent for CrPV, HCV, and c-Myc IRESes, while EMCV IRES translation was not affected by changes in the methylation of this specific site (Figure 5F). These results might be explained by the augmented ribosome affinity for IRESes secondary structures, which was probably caused by changes in the ribosome structure mediated by C2848 methylation status.

## 3. Experimental Section

### 3.1. Patient Materials

Both tumor and adjacent non-tumor (hereinafter referred to as “normal tissue”) colon tissues selected from a series of thirty-four consecutive patients who had undergone surgical resection for primary carcinoma at the Surgical Department of the University of Bologna, on the sole basis of frozen tissue availability. For each patient, clinical information was recorded and the corresponding tissue was histologically characterized and processed following standard procedures to define bio-pathological features. Specimen collection and tissue analyses were approved by the Bologna University Ethical Committee on human tissue research. Tissues were preserved at −80 °C until use. A 60 mg piece for each sample was minced in liquid nitrogen and then lysed for total RNA extraction using Tri-reagent solution (Ambion, Life Technologies Corporation, Monza, Italy) according to the manufacturer’s instructions.

### 3.2. Cell Cultures and Treatments

All colon cancer cell lines were grown in their optimal culture medium and conditions at 37 °C in 5% CO_2_ according to the American Type Culture Collection instructions.

Lymphocytes were recovered from healthy donors’ buffy coats collected by the transfusion center of Sant’Orsola-Malpighi Hospital in Bologna after ficoll gradient and monocyte depletion. Human lymphocytes were grown in RPMI-1640 medium (Sigma, Milan, Italy), supplemented with 10% FBS, 1% of penicillin and streptomycin, 1% of glutamine, and 0.5% of non-essential amino acids (all Sigma). Lymphocyte proliferation assays were performed by adding 10 μg/mL of phytohemoagglutinin (PHA) (Sigma) to the medium.

The inhibition of ribosome biogenesis and mRNA stability assay were performed by exposing cells to Actinomycin D (Act-D) (Sigma) at a concentration of 8 nM and 80 nM, respectively, and assessed at different time points (20 min, 1 h, 3 h, 8 h, and 24 h of treatments). SW620 cells were seeded at 100,000 cells/well in 6-well plates and treated with Act-D, while human lymphocytes were seeded at 1,000,000 cells/mL, PHA stimulated for 72 h (where necessary), and then treated with Act-D. PHA stimulation was evaluated by cell counting and considered effective only when lymphocytes proliferation was increased more than 5 fold compared to control cells.

### 3.3. Gene Expression Assays

Total RNA was extracted from cell lines using Tri-reagent (Ambion) and 1 μg was reverse-transcribed using the High-Capacity cDNA Archive Kit (Applied Biosystems, Carlsbad, CA, USA). Real-time PCR analysis was carried out in a Gene Amp 7000 Sequence Detection System (Applied Biosystems) using the TaqMan approach for β-glucuronidase (GUS) (Applied Biosystems) and SYBR green approach for all other genes, using specific couples of primers. The housekeeping GUS gene was used to calculate the relative amounts of the studied target genes in all experiments except for those in which Act-D treatment was performed. In the latter case, for each sample we calculated the 2^^−^*^Ct^*, assumed the values of untreated cells as reference (100%), and then calculated the relative percentage of investigated genes. For each sample, three replicates were analyzed. Analyses in cell lines and tumors were carried out by using aliquots of a single stock of cDNA obtained from HCT116 as internal calibrator. The analysis of U50 levels was performed by qPCR using a primer with a linker sequence attached to a U50-specific sequence for cDNA synthesis and reverse linker specific primer, and U50 forward specific primer for real-time PCR as described in Dong, 2008 [21]. All primers used were reported in Table S2.

### 3.4. SnoRNA U50 Knockdown and Upregulation

Since it has been shown that RNAi is an unsuitable tool for snoRNA KD [30], we used an oligomediated RNaseH cleavage strategy. A custom-made hybrid DNA-RNA Locked Nucleic Acid (LNA)-antisense specific for U50 (Exiqon, Vedbaek, Denmark) was transfected into cells with Lipofectamine RNAiMAX reagent (Invitrogen, Life Technologies, Carlsbad, CA, USA), while the efficiency of KD was evaluated after 24 h and 72 h. The same amount of a control oligo sequence (TCGAGCGGCCGCCCGGGC) was transfected with lipofectamine in control cells.

For U50 upregulation, we used a retroviral transduction system. We briefly transfected the Phoenix A cell line with 10 μg of pSIREN-RetroQ-U50 plasmid or the pSIRENRetroQ vector control (gifts of Prof. Dong) [21] using Lipofectamine 2000 reagent, and collected the supernatant (containing viruses) after 24 h and 48 h. After that we infected HCT116, SW620, and LoVo cell lines previously seeded in 6-well plates with viruses using standard spinoculation protocols. Seventy-two hours after infection, cell lines were selected for at least 10 days, while adding puromycin into the media at a final concentration of 0.7 μg/mL. The upregulation of U50 was verified by qPCR as described above.

### 3.5. Semi-Quantitative Site-Specific Methylation Assay

To evaluate site-specific (C2848) methylation levels, we followed a modified previously described method [19]. This modified method consists of the reverse transcription (RT) of total RNA with Moloney Murine Leukemia Virus Reverse Transcriptase (M-MLV) by using a 28S specific oligonucleotide (targeting the methylation site downstream, at either low or high dNTPs concentrations, either affecting cDNA synthesis or not, respectively, in the presence of modified sites. The cDNAs resulting from both RT are evalutated by qPCR; the ratio between high and low dNTPs-derived products semi-quantitatively indicates the modification of the site downstream of the oligonucleotide used. An endogenous housekeeping RNA (GUS) was used to evaluate the efficiency of reverse transcription.

### 3.6. mRNA Transfection and Internal Ribosome Entry Site (IRES) Translation

Capped mRNA was transcribed from linearized pR-CrPV-IRES-F (gift of Dr. D. Ruggero) [31], pF-EMCV-IRES-R (gift of Prof. A.C. Palmenberg) [32], pR-HCV-IRES-F (gift of Prof R.E. Lloyd) [33], and pR-c-MYC-IRES-F [34], by using the mMessage mMachine T7 or T3 kits (Ambion). Cells were transfected with 0.4 μg RNA/sample using Lipofectamine 2000 (Invitrogen) following the manufacturer’s instructions. After 4 h transfection, media were changed and 2 h later cells were harvested and analyzed with a dual-luciferase assay kit (Promega, Madison, WI, USA) according to the manufacturer’s instructions.

### 3.7. Statistical Analysis

All data were analyzed using Prism software, version 5.0a. A paired *t*-test was used for patient data set when comparing normal and correspondent tumoral tissues. A two-sided Student’s *t*-test was used for the comparisons between two groups. *p* values <0.05 were regarded as statistically significant.

## 4. Conclusions

Recent reports have demonstrated that U50 was downregulated in prostate and breast cancers and in B-cell lymphomas [20,21,23]. In this study we have demonstrated for the first time that snoRNA U50 expression: (i) is also downregulated in colon cancer; (ii) is reduced during cell proliferation; and (iii) its levels are inversely associated with ribosome biogenesis. Furthermore, since U50 mediates site-specific methylation on C2848 of 28S rRNA, we investigated the effect of its modulation on ribosome activity and showed that proliferating cells with low levels of both U50 and C2848 methylation only moderately changed their translational activity, while the U50 and C2848 methylation increments brought about an IRES-mediated translation propensity. At the present time the biological significance of these observations in cancer progression is unclear, and further investigations will aim to investigate which genes are differently expressed and what their roles might be in human cancers and other pathologies.

## Supplementary Information
