# Supplementary material for: SnoRNA U50 Levels Are Regulated by Cell Proliferation and rRNA Transcription
Source: Int J Mol Sci. 2013 Jul 17;14(7):14923–35. doi: 10.3390/ijms140714923 (PMC3742280; doi:10.3390/ijms140714923)
Supplement: Supplementary file 1 [file ijms-14-14923-s001.pdf]

Supplementary Information

Table S1. Clinical and biopathological features of patients and cancer tissues.

Features	*N* (34 total)
**Age (years) (mean ± SD): 74 ± 11**	
**Tumor localization**	
Left Colon	15
Right Colon	19
**Diameter (mm) (mean ± SD): 45 ± 16**	
**Tumor size (pT classification)**	
T1	1
T2	2
T3	29
T4	2
**Lymph Node status (pN)**	
N0	20
N1	7
N2	7
**Grade (G)**	
Low	27
High	7
**Tumor stage**	
I and II	20
III and IV	14
**Ki67 expression (%)**	
20	32
20	2

Table S2. List of primers sequences.

LNA	5'-TTTACGGATCTGGCTTCT-3'
U50 specific RT primer	5'-TCGAGCGGCCGCCCGGGCAGGTATCTCAGAAGCCAGATCCG-3' (Underlined as linker sequence)
U50 F	5'-TATCTGTGATGATCTTATCCCGAACCTG AAC-3'
U50 R	5'-TCGAGCGGCCG CCCGGGC-3'
28S specific RT primer	5'-GCCCCGCTTCGCGCCCCAGC-3'
28S F	5'-AGGTAAGGGAAGTCGGCAAG-3'
28S R	5'-CAGCCCTTAGAGCCAATCCT-3'
SNHG5 F	5'-TAGAGATGCAAAGATACACGAAA-3'
SNHG5 R	5'-CACACTCAGAACGCTGTTCAC-3'
U33 F	5'-TCCCACTCACATTCGAGTTTC-3'
U33 R	5'-GGCCTCAGATGGTAGTGCAT-3'
U34 F	5'-CATGATGTTCCGCAACTACCTA-3'
U34 R	5'-GTCTCAGCCAGTGCTGCTTT-3'
U56 F	5'-TAGTCTGGCTCGTGGGACTC-3'
U56 R	5'-GGCCTCAGATGGTAGTGCAT-3'

Figure S1. Relationship between SNHG5 and rRNA transcription in proliferating lymphocytes. Evaluation of U50 host gene levels (SNHG5) in PHA stimulated cells after blockade of Polymerase I and II activities with Act-D 80 nM. The results correspond to means ± S.E.M. of three different experiments. PHA, phytohemagglutinin; Act-D, actinomycin-D.

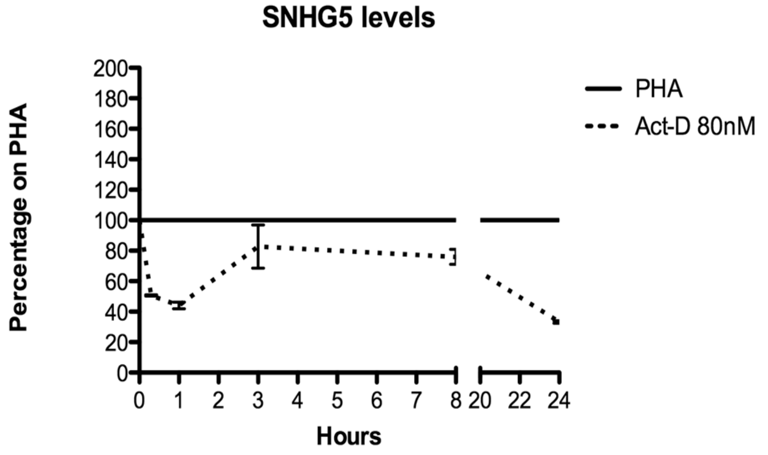

Figure S2. Relationship between GUS and SNHG5 mRNAs and *de novo* rRNA transcription in SW620 cell line. Evaluation of GUS and SNHG5 transcripts in SW620 cell line after blockade of only Polymerase I with Act-D 8 nM. The results correspond to means ± S.E.M. of three different experiments. CTR, controls; Act-D, actinomycin-D.

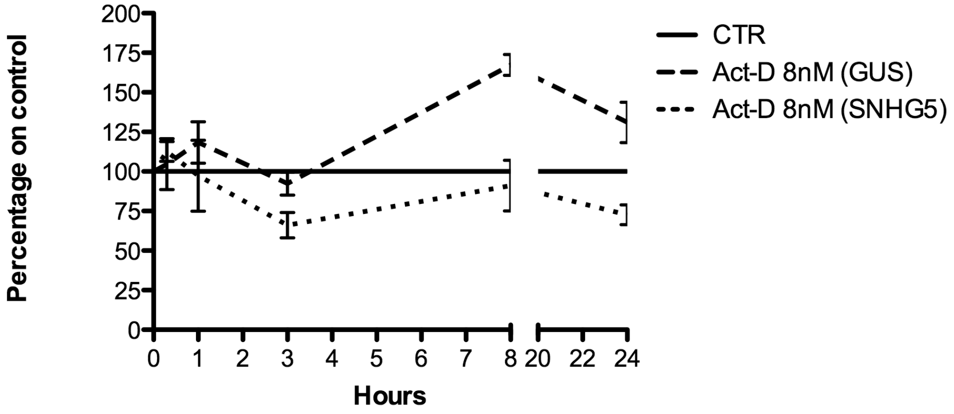

Figure S3. Relationship between C/D box snoRNAs U33, U34, U56 and *de novo* rRNA transcription in SW620 cell line. Evaluation of C/D box snoRNAs U33, U34 and U56 stability performed by measuring their levels and 45S in SW620 cell line after blockade of Polymerase I with low dose of Act-D. The analyses were performed after 20 min, 1 h, 3 h, 8 h and 24 h after Act-D treatment. The results correspond to means ± S.E.M. of three different experiments. CTR, controls; Act-D, actinomycin-D.

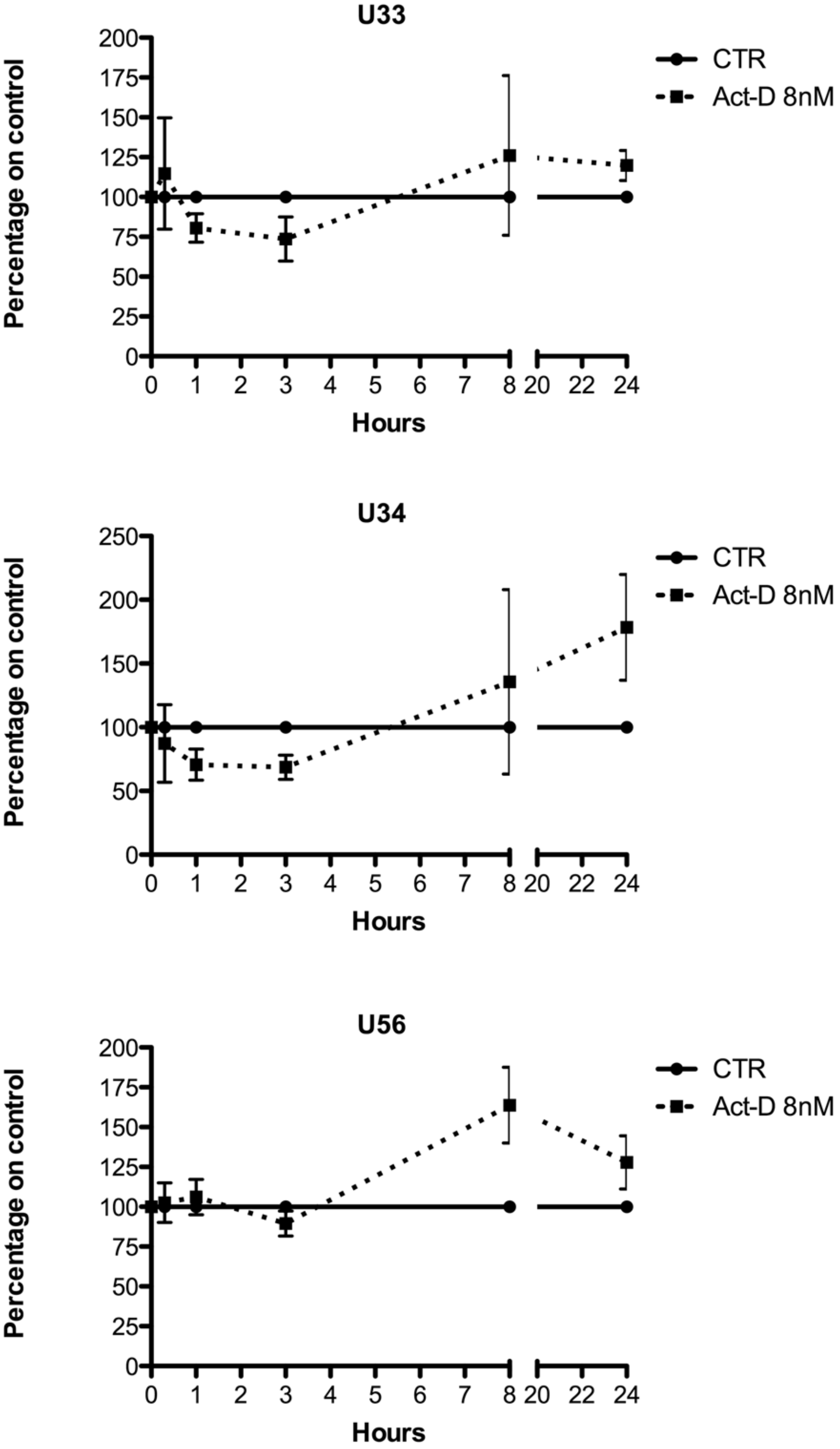

Figure S4. Analysis of C2848 methylation levels. Analysis of C2848 methylation status of 28S rRNA on human lymphocytes stimulated to proliferate with PHA. Since U50 guides the methylation reaction on this specific site, the observed reduction was in line with the reduction of U50 after PHA-treatment. The results correspond to means ± S.E.M. of three different experiments. *** $p \leq 0.001$; CTR, controls; PHA, phytohemagglutinin.

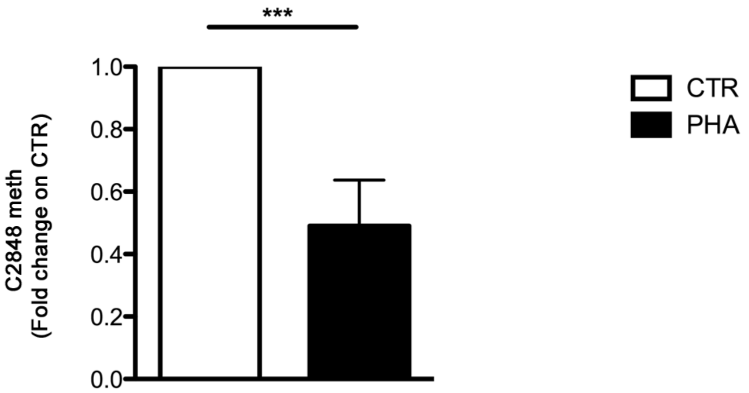

Figure S5. Effect of U50 modulation on Cap- and IRES-dependent translation. Evaluation of Cap- and IRES-dependent translation on HCT116 after U50 KD (top) and overexpression (bottom). The analyses were carried out evaluating luciferase activities of transfected bi-cistronic mRNAs containing CrPV, EMCV, HCV and c-myc IRESes. The results correspond to means ± S.E.M. of three different experiments. CTR, controls; KD, knockdown; pSrQ, pSIREN-RetroQ vector (empty vector); pSrQ U50, pSIREN-RetroQ-U50 vector; IRES, Internal Ribosome Entry Site; CrPV, CRicket Paralysis Virus; EMCV, EncephaloMyoCarditis Virus; HCV, Hepatitis C Virus.

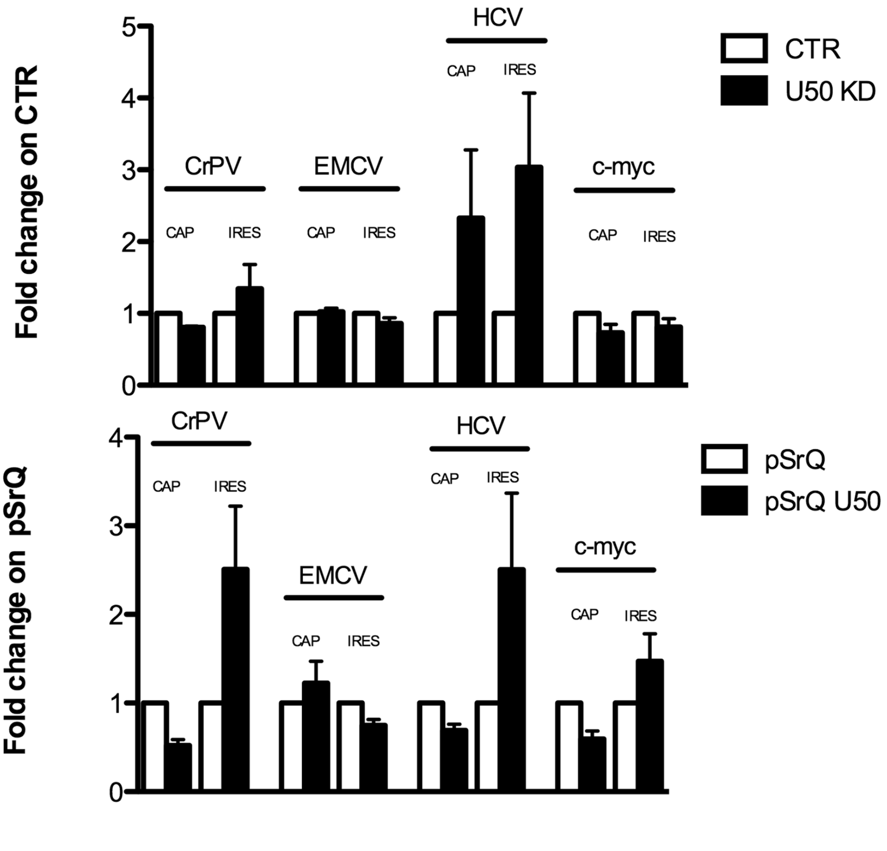

Figure S6. U50 overexpression on SW620 and LoVo cells. Analysis of U50 and C2848 methylation levels in SW620 and LoVo cells overexpressing U50. The increase in U50 levels did not affect C2848 methylation status. The results correspond to means ± S.E.M. of three different experiments. pSrQ, pSIREN-RetroQ vector (empty vector); pSrQ U50, pSIREN-RetroQ-U50 vector.

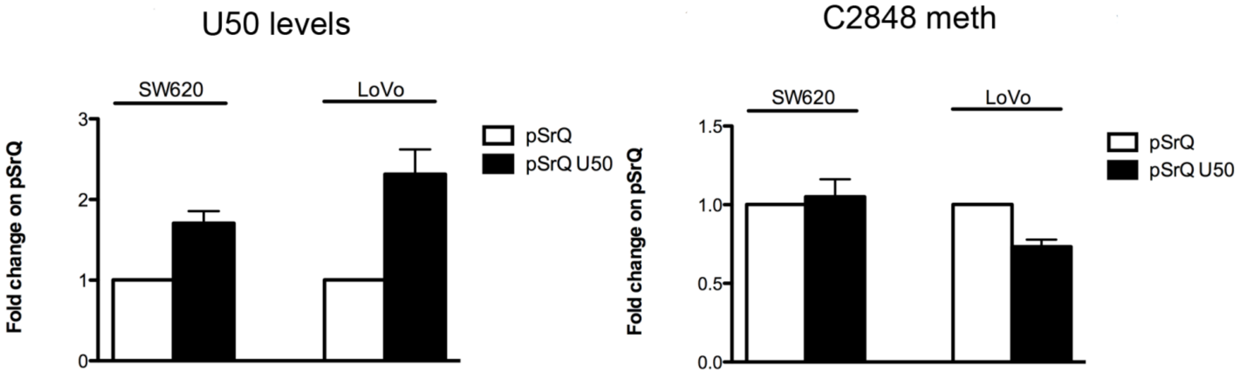

© 2013 by the authors; licensee MDPI, Basel, Switzerland. This article is an open access article distributed under the terms and conditions of the Creative Commons Attribution license (http://creativecommons.org/licenses/by/3.0/).
